# Advances in Research on *Dioscorea nipponica* Makino: Chemical Constituents, Biological Activities and Developmental Prospects

**DOI:** 10.3390/molecules31142460

**Published:** 2026-07-14

**Authors:** Li Yuan, Yang-En Sun, Ya-Peng Liang, Da-Hong Yao, Ya-Ping Guo, Xun Song, Zhen-Dan He, Bing Zhao

**Affiliations:** 1 College of Pharmacy, Shenzhen Technology University, No. 3002, Lantian Road, Pingshan District, Shenzhen 518118, China; 2410265071@stumail.sztu.edu.cn (L.Y.); yaodahong@sztu.edu.cn (D.-H.Y.); guoyaping@sztu.edu.cn (Y.-P.G.); songxun@sztu.edu.cn (X.S.); 2 Shandong Key Laboratory of Gelatine Medicines Research and Development, Dong’e Ejiao Co., Ltd., No. 78, Ejiao Street, Liaocheng 252201, China; sunye@dongeejiao.com (Y.-E.S.); liangyapeng@dongeejiao.com (Y.-P.L.)

**Keywords:** *Dioscorea nipponica*, steroidal saponins, anti-inflammatory activity, traditional medicine

## Abstract

Ethnopharmacological relevance: *Dioscorea nipponica* Makino is a traditional medicinal plant widely used in East Asia for the treatment of rheumatic disorders, inflammatory diseases, and cardiovascular conditions. Its rhizome has long been applied in clinical practice for relieving pain, promoting blood circulation, and reducing swelling. Aim of the study: This narrative review aims to provide a comprehensive and critical overview of the phytochemical constituents, pharmacological activities, and underlying mechanisms of *D. nipponica* and to identify current research gaps and future perspectives. Materials and methods: The literature was searched in PubMed, Web of Science, ScienceDirect, CNKI and Wanfang Data from database inception to December 2025. The combined retrieval keywords were set as: (*Dioscorea nipponica* Makino OR Chuanshanlong) AND (chemical constituents OR steroidal saponins OR flavonoids OR phenols) AND (biological activity OR anti-inflammatory OR cardioprotective OR hepatotoxicity OR clinical application). Both English and Chinese publications were retrieved, and studies written in other languages were excluded. Results: Phytochemical studies have identified diverse secondary metabolites, particularly steroidal saponins, along with diarylheptanoids and phenanthrenes. These compounds exhibit multiple pharmacological activities, including anti-inflammatory, anti-tumor, immunomodulatory, and cardioprotective effects. Mechanistic studies indicate that these activities are mediated through the modulation of key signaling pathways such as NF-κB, PI3K/Akt, AMPK, and the NLRP3 inflammasome. However, current research remains fragmented, with limited integration of chemical composition, molecular targets, and therapeutic outcomes. Conclusions: *D. nipponica* represents a promising source of bioactive natural products, with steroidal saponins as the major contributors to its pharmacological effects. Future studies should focus on multi-component interactions, pharmacokinetics, quality control, and clinical validation to support its rational development and sustainable utilization.

## 1. Introduction

**Figure 1:**
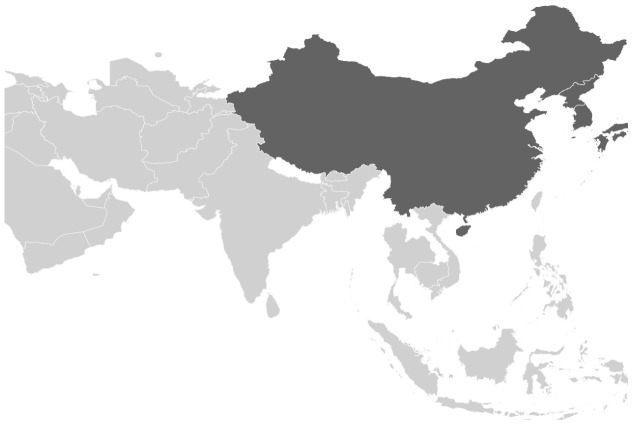
Geographic distribution of *Dioscorea nipponica* in East Asia. The species is mainly distributed in China, Japan, North Korea and South Korea, which are highlighted in dark grey.

*Dioscorea nipponica* Makino, commonly known as “Chuan-shan-long” in China, is a perennial twining herb belonging to the family Dioscoreaceae. It is primarily distributed in northeastern, northern, eastern, and central China, where it grows in humus-rich mountain forest soils at altitudes ranging from 100 to 1800 m [1]. Its geographical distribution across East Asia is illustrated in Figure 1. The plant is characterized by a cylindrical, lignified rhizome and cordate leaves. The rhizome is the officially recognized medicinal part and is listed in the Chinese Pharmacopoeia as “*Dioscoreae Nipponicae* Rhizoma” [2].

Phytochemical studies have revealed that *D. nipponica* contains diverse classes of secondary metabolites. Among them, steroidal saponins are the most abundant and extensively investigated constituents and are generally considered to be the main bioactive components of this plant [3,4]. In addition to steroidal saponins, other compounds such as diarylheptanoids, phenanthrene derivatives, and several minor metabolites have also been identified [5,6,7]. Pharmacological investigations have demonstrated that extracts and isolated constituents of *D. nipponica* exhibit a variety of biological activities, including anti-inflammatory, anti-tumor, antioxidant, immunomodulatory, and cardioprotective effects [1,8]. These findings provide scientific support for many of its traditional medicinal uses and highlight the potential value of this plant as a source of bioactive natural products.

Despite increasing research interest, a comprehensive synthesis of the biological activities, phytochemical composition, and practical applications of *D. nipponica* remains lacking. The present review addresses this gap by systematically summarizing and analyzing relevant studies retrieved from PubMed and Google Scholar. It provides an updated overview of the plant’s botany, ethnomedicinal uses, pharmacological activities, phytochemical constituents, and clinical applications. This analysis is intended to support further research and rational utilization of this medicinal species.

## 2. Botanical Characteristics

### 2.1. Morphology

**Figure 2:**
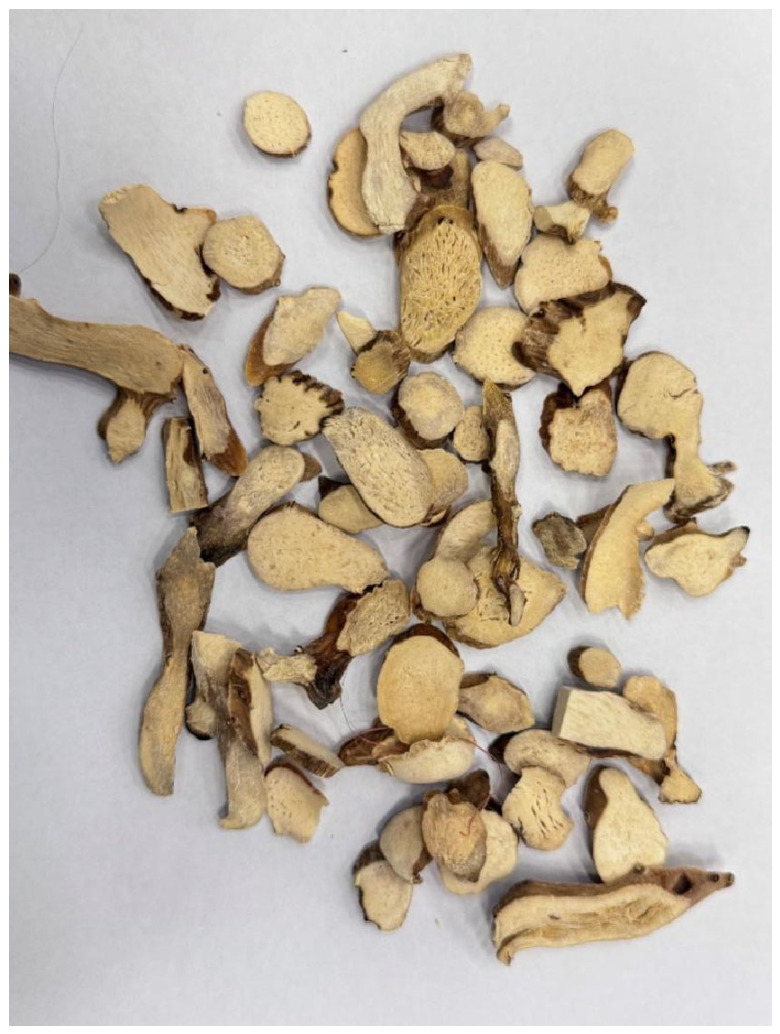
Photo of the rhizomes of *D. nipponica*.

Botanically, *D. nipponica* is a perennial twining herb that can reach up to 5 m in length. The rhizome is transverse, cylindrical, woody, and highly branched, with a conspicuously exfoliating cork layer [9]. Stems are left-twining, cylindrical, and nearly glabrous. Leaves are simple and alternate, with petioles 10–20 cm long. The leaf blades are palmately cordate and highly variable in morphology. The adaxial surface is yellowish-green and glossy and may be glabrous or sparsely covered with fine white hairs that are denser along the veins [10]. Flowers are unisexual, and the species is dioecious [11]. The rhizome is slightly curved, measuring approximately 15–20 cm in length and 1.0–1.5 cm in diameter. Its external surface ranges from yellowish-white to brownish-yellow, with irregular longitudinal furrows, residual spinous rootlets, and laterally projecting stem scars. The texture is firm, and the fracture surface is smooth and even, appearing white to yellowish-white with scattered light-brown vascular bundle points. The rhizome possesses a faint odor and a distinctly bitter and astringent taste. The morphological characteristics of its rhizome are displayed in Figure 2.

### 2.2. Growth and Development

*D. nipponica* inhabits cool-temperate deciduous forests of the Sino-Japanese floristic region at elevations of 400–1800 m. It typically grows in humus-rich sandy loam soils with a pH of 5.5–6.5, under 60–80% canopy cover, with annual precipitation of 700–1200 mm and mean temperatures ranging from 4 to 12 °C [12]. As a hemicryptophyte, the aerial parts wither each autumn, while the rhizome undergoes apical thickening accompanied by saponin accumulation. The diosgenin content increases from approximately 0.5–0.8% in the first year to 2.5–3.5% after 4–5 years of growth. Sexual reproduction is relatively limited, with seed germination rates below 20% following 12–16 weeks of cold stratification [13]. Consequently, commercial cultivation relies primarily on vegetative propagation using approximately 3 cm rhizome fragments, achieving establishment rates of 85–90%. However, prolonged clonal propagation may reduce genetic diversity and potentially affect the quality of wild germplasm resources.

## 3. Traditional Use

For more than 2000 years, the dried rhizome of *Dioscorea nipponica* has been used in traditional Chinese medicine among the Miao, Mongol, and Han ethnic groups. According to Jiang-Su-Yi-Yao and other classical materia medica records, the crude drug is described as bitter-sweet in flavor and warm in nature and is believed to act on the liver, lung, and kidney meridians. Traditionally, it has been administered as a decoction or soaked in rice wine to dispel wind-dampness and alleviate rheumatic pain (Bi syndrome), promote collateral circulation, and reduce swelling of the lower limbs and lumbar region. It has also been used to relieve cough and asthma symptoms, resolve phlegm in chronic bronchitis, promote blood circulation, and facilitate the healing of bruises, sprains, and fractures [2].

For external use, the fresh rhizome was traditionally pounded into a paste and applied to treat carbuncles, skin ulcers, and traumatic injuries [14]. In some folk medical practices, the rhizome was also used for the treatment of limb numbness, lower back pain, and joint stiffness associated with chronic rheumatic conditions [1,15]. These traditional applications have provided the basis for the development of modern *Dioscorea nipponica*-containing preparations, such as “Gulong Jiaonang” capsules and “Chuan-long-tong-mai” pills, which are used in the management of rheumatoid arthritis.

In addition to its use in traditional Chinese medicine, *D. nipponica* has also been employed in regional folk medicine systems in northern and northeastern China. Ethnopharmacological records indicate that the rhizome has been used to improve blood circulation, relieve pain, and reduce inflammation in musculoskeletal disorders [16]. In some traditional practices, it was also incorporated into herbal formulations for treating respiratory disorders, particularly chronic cough and asthma accompanied by excessive phlegm [1]. These long-standing traditional uses have attracted increasing scientific interest and have provided important clues for modern pharmacological investigations into the bioactive constituents and therapeutic potential of this medicinal plant.

## 4. Chemical Composition

**Figure 3:**
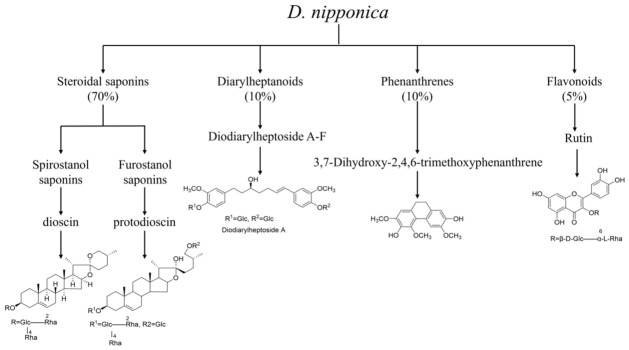
Classification of major chemical constituents of *D. nipponica*.

The main chemical constituents of the rhizome of *Dioscorea nipponica* are steroidal saponins, while its aerial parts are primarily composed of phenanthrenes, phenanthrenequinones, and related compounds. The classification of its main chemical components is summarized in Figure 3.

### 4.1. Steroidal Saponins

Steroidal saponins are recognized as the major bioactive constituents of *D. nipponica*, with reported content varying depending on origin, harvest time, and processing methods. Quantitative analyses based on chromatographic techniques, including HPLC and UPLC-MS, have shown that the total saponin content in rhizome samples typically ranges from approximately 2–12% (*w*/*w*) [17,18], although considerable variation exists among different studies. The reported contents of major steroidal saponins in *D. nipponica* vary across studies due to differences in geographic origin, harvest conditions, and analytical methods. Based on multiple chromatographic studies, protodioscin, protogracillin, methylprotodioscin, pseudoprotodioscin, dioscin, and gracillin are consistently identified as representative compounds [17]. A summary of their reported ranges and analytical methods is provided in Table 1.

#### 4.1.1. Spirostanol Saponins

**Figure 4:**
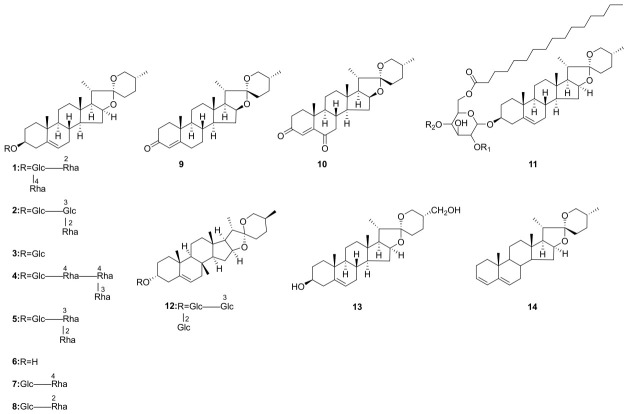
Chemical structures of spirostanol saponins in *D. nipponica* (Glc = β-*D*-glucopyranosyl, Rha = α-*L*-rhamnopyranosyl).

Kang et al. [22] isolated the spirostanol saponins dioscin and gracillin from the total steroidal saponin fraction of *D. nipponica*. Li et al. [23] identified trillin in the methanol extract of *D. nipponica* using reverse-phase high-performance liquid chromatography. Du et al. [24] isolated dioscin Dc and Diosgenin-3-O-{α-*L*-rhamnopyranosyl-(1→2)-[α-*L*-rhamnopyranosyl-(1→3)]}-β-*D*-glucopyranoside from the total saponins of *D. nipponica*. Zhang et al. [25] isolated 25-*D*-spirosta-3,5-diene and 3-O-[α-*L*-rhamnopyranosyl(1→2)-{α-*L*-rhamnopyranosyl(1→4)}-(6’-O-hexadecanoyl)-β-*D*-glucopyranosyl]-25-(*R*)-spirost-5-en-3-β-ol from the water-soluble part of *D. nipponica*. Shu et al. [26] isolated diosgenin, Progenin II, Progenin III, Diosgenone, Diosgenin-3,6-dione from the 60% ethanol extract of *D. nipponica*. Chakravarti, D. et al. [27] isolated smilagenone from the long-term water extract of *D. nipponica*. Structures are shown in Figure 4.

#### 4.1.2. Furostanol Saponins

**Figure 5:**
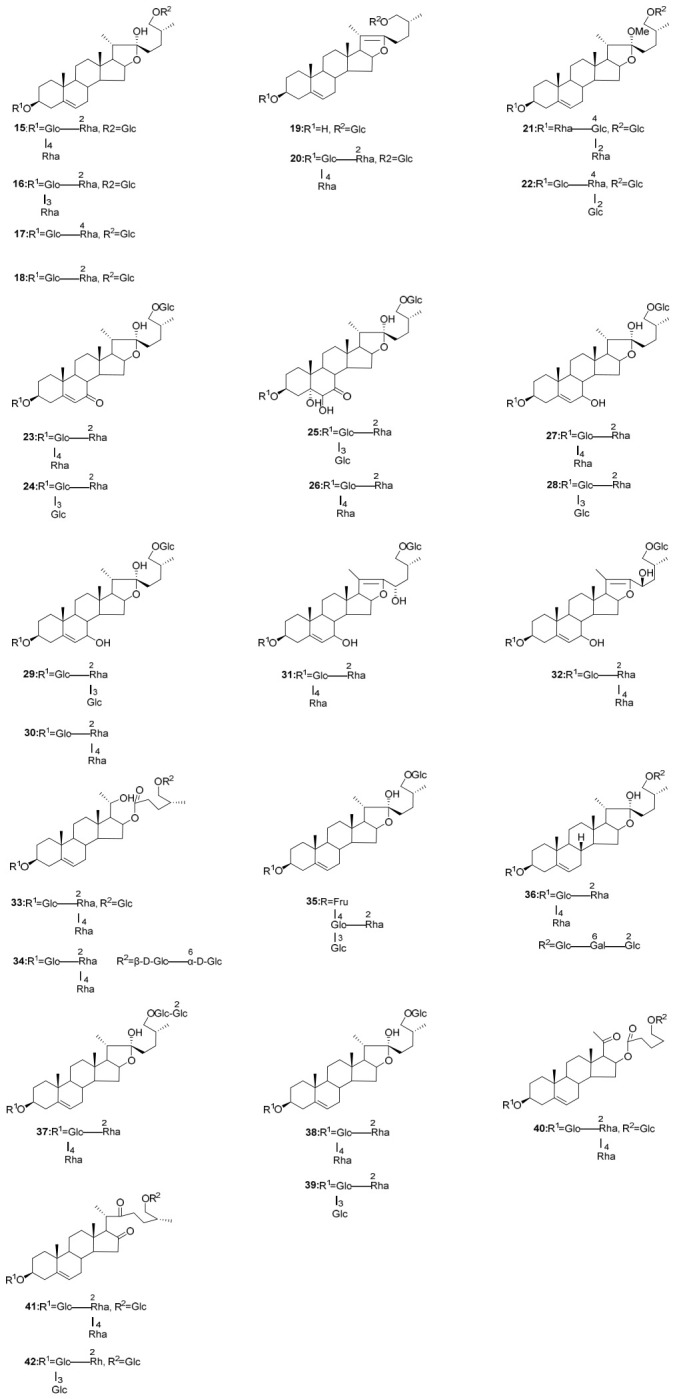
Chemical structures of furostanol saponins in *D. nipponica* (Glc = β-*D*-glucopyranosyl, Rha = α-*L*-rhamnopyranosyl).

Kang et al. [22] isolated the spirostanol saponins protodioscin and protogracillin from the total steroidal saponin fraction of *D. nipponica*. Shu et al. [26] isolated methylprotodioscin from the 60% ethanol extract of *Dioscorea nipponica*. Tang et al. [28] identified kikuba-saponin from *D. nipponica* by UPLC-QTOF-MS analysis. Cui et al. [29] isolated 3β,26-dihydroxy-25(*R*)-furosta-5,20-dien-26-O-β-*D*-glucopyranoside from the aqueous extract of *D. nipponica*. Dong et al. [30] isolated pseudoprotodioscin from the rhizomes of *D. nipponica*. Zhang et al. [31] identified methylprotogracillin from *D. nipponica*. Liu et al. [32] isolated (25*R*)-26-O-β-*D*-glucopyranosyl-furost-5(6)-en-3β,22α,26-triol-3-O-[α-*L*-rhamnopyranosyl-(1→4)]-β-*D*-glucopyranoside, Diosnipponicoside A, Diosnipponicoside B, Diosnipponicoside C, Diosnipponicoside D, Diosnipponicosid E, Diosnipponicoside F, Diosnipponicoside G, Diosnipponicoside H, Diosnipponicoside I, Diosnipponicoside J, Diosnipponicoside K, Diosnipponicoside L, Diosnipponicoside M, Diosnipponicoside N, Diosnipponicoside O, 25R-Dracaenoside O, 25*R*-Dracaenoside P, hypoglaucin G, anguivioside XV, and 3-O-[α-*L*-rhamnopyranosyl-(1→2)]-[β-*D*-glucopyranosyl-(1→3)]-β-*D*-glucopyranosyl-26-O-β-*D*-glucopyranosyl-cholest-5-en-16,22-dione from the 70% ethanol extract of *Dioscorea nipponica*. Structures are shown in Figure 5.

### 4.2. Diarylheptanoids

**Figure 6:**
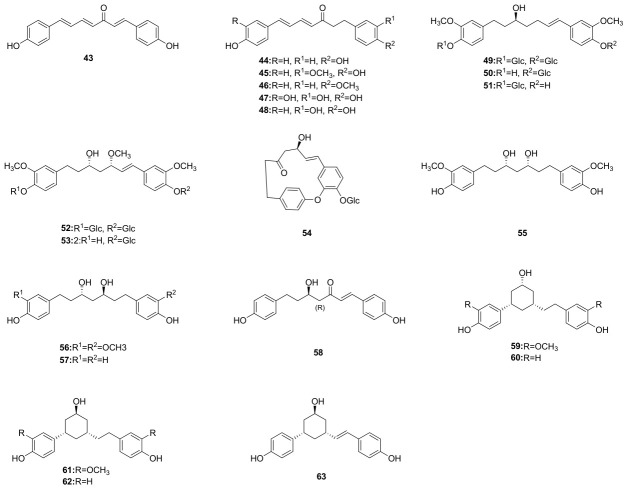
Chemical structures of diarylheptanoids in *D. nipponica* (Glc = β-*D*-glucopyranosyl).

Lu et al. [6] isolated 1,7-bis(4-Hydroxyphenyl)-1,4,6-heptatrien-3-one and 1,7-bis(4-Hydroxyphenyl)-4,6-heptabien-3-one from the aerial parts of *D. nipponica*. Meng et al. [7] isolated tsaokoarylone, diodiarylheptoside A, diodiarylheptoside B, diodiarylheptoside C, diodiarylheptoside D, diodiarylheptoside E, diodiarylheptoside F, (+)-hannokinol, (3*S*,5*S*)-3,5-dihydroxy-1,7-bis-(4-hydroxy-3-methoxyphenyl)heptane, and (3*R*,5*S*)-3,5-dihydroxyl-1,7-bis-(4-hydroxy-3-methoxyphenyl)heptane from the rhizomes of *D. nipponica*. Woo, K. W. et al. [33] isolated 1,7-bis(4-hydroxyphenyl)hepta-4*E*,6*E*-dien-3-one, 1,7-bis(3,4-dihydroxyphenyl)hepta-4*E*,6*E*-dien-3-one, (4*E*,6*E*)-1-(3’,4′-dihydroxyphenyl)-7-(4″-hydroxyphenyl)-hepta-4,6-dien-3-one, (4′-hydroxyphenyl)-7-(4″-hydroxyphenyl)-hepta-1-en-3-one, diosniponol A, diosniponol B, (1*S*,3*R*,5*S*)-1,7-bis(4-hydroxyphenyl)-1,5-epoxy-3-hydroxyheptane, (1*S*,3*S*,5*S*)-1,7-bis(4-hydroxyphenyl)-1,5-epoxy-3-hydroxyheptane, and (1*S*,3*S*,5*R*,6*E*)-1,7-bis(4-hydroxyphenyl)-1,5-epoxy-3-hydroxy-hept-6-one from the rhizomes of *D. nipponica*. Structures are shown in Figure 6.

### 4.3. Phenanthrenes and Phenanthrenequinones

**Figure 7:**
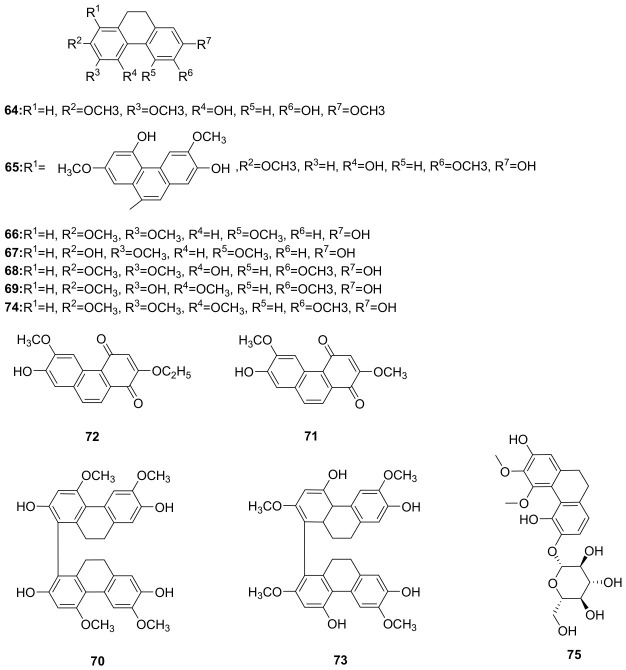
Chemical structures of phenanthrenes and phenanthrenequinones in *D. nipponica*.

Lu et al. [34] isolated 4,6-Dihydroxy-2,3,7-trimethoxy-9,10-dihydrophenanthrene, 1-(4,7-Dihydroxy-2,6-dimethoxy-9,10-dihydrophenanthrenyl)-4,7-dihydroxy-2,6-dimethoxy-9,10-dihydrophenanthrene, 7-Hydroxy-2,3,5-trimethoxy-9,10-dihydrophenanthrene, 2,7-Dihydroxy-3,5-dimethoxy-9,10-dihydrophenanthrene, 4,7-Dihydroxy-2,3,6-trimethoxyphenanthrene, 3,7-Dihydroxy-2,4,6-trimethoxyphenanthrene, 1-(2,7-Dihydroxy-4,6-dimethoxyphenanthrenyl)-2,7-dihydroxy-4,6-dimethoxyphenanthrene, 7-Hydroxy-2,6-dimethoxy-1,4-phenanthraquinone, 2-ethoxy-7-hydroxy-6-methoxy-1,4-phenanthraquinone, 7-Hydroxy-2,3,5-trimethoxy-9,10-dihydrophenanthrene, and 2,7-dihydroxy-3,4,6-trimethoxy-9,10-dihydrophenanthrene. Liu et al. [32] isolated diosniposide B from the 70% ethanol extract of *D. nipponica*. Structures are shown in Figure 7.

### 4.4. Phenols, Organic Acids and Amino Acids

**Figure 8:**
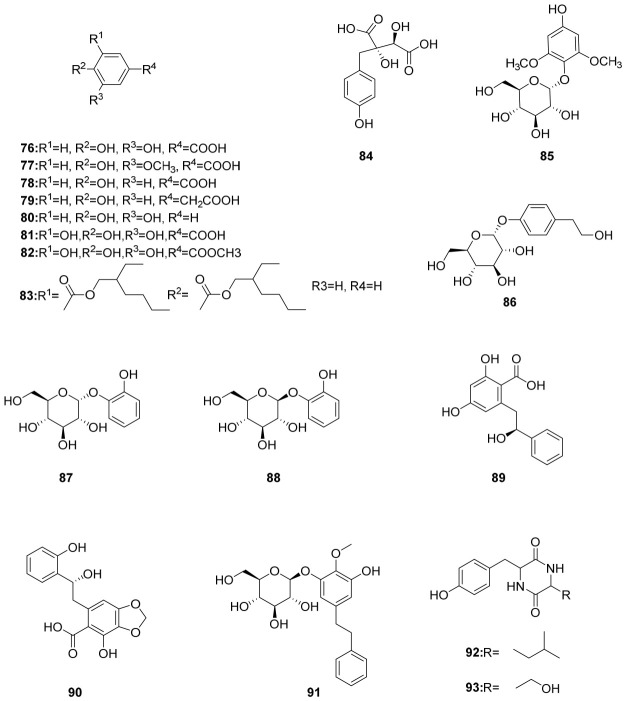
Chemical structures of phenols, organic acids and amino acids in *D. nipponica*.

Lu et al. [6] isolated protocatechuic acid, vanillic acid, 4-Hydroxybenzoic acid, and Pyrocatechol from the stems and leaves of *D. nipponica*. Xia et al. [4] isolated gallic acid and methyl gallate from the dried rhizomes of *D. nipponica*. Chen et al. [35] identified hydroxyphenylacetic acid in the lipophilic fraction of the aerial parts of *D. nipponica* using GC-MS. Zhang et al. [25] isolated benzoic acid, 4-hydroxy-2,6-dimethoxyphenyl-β-*D*-glucopyranoside, 2-(4-hydroxyphenyl)-ethyl-β-*D*-glucopyranoside, Pyrocatechol 1-O-β-*D*-glucopyranoside, Pyrocatechol 1-O-α-*D*-glueopyranoside, cyclo-(Leu-Tyr), and cyclo-(Ser-Tyr) from the water-soluble fraction of *D. nipponica*. He et al. [36] isolated and identified piscidic acid from the aqueous extract of *D. nipponica*. Liu et al. [32] isolated diosniposide A, diosniponol C and diosniponol A from the 70% ethanol extract of *D. nipponica*. Structures are shown in Figure 8.

### 4.5. Flavonoids

**Figure 9:**
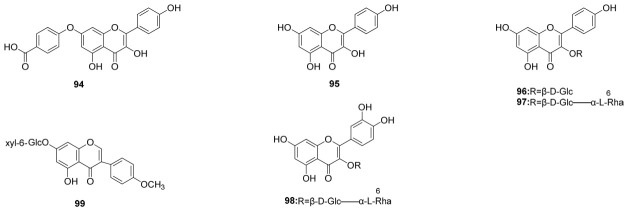
Chemical structures of flavonoids in *D. nipponica* (Glc = β-*D*-glucopyranosyl, Rha = α-*L*-rhamnopyranosyl, xyl = β-*D*-xylopyranosyl).

Chen et al. [37] identified Kaempferol-7-O-benzoate and Kaempferol in the lipophilic fraction of the aerial parts of *D. nipponica* by GC-MS. Lu et al. [5] isolated kaempferol-3-O-β-*D*-glucopyranoside, kaempferol-3-O-β-rutinoside, and rutin from the aerial parts of *D. nipponica*. Liu et al. [32] isolated kushenol O from the 70% ethanol extract of *D. nipponica*. Structures are shown in Figure 9.

### 4.6. Steroids

**Figure 10:**
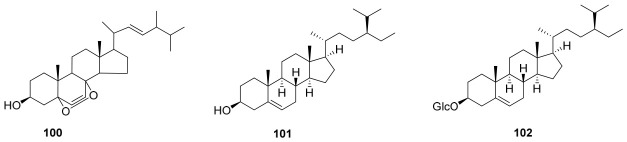
Chemical structures of steroids in *D. nipponica* (Glc = β-*D*-glucopyranosyl).

Chen et al. [6,37] identified ergosterolperoxide, β-sitosterol, and claucosterol in the lipophilic fraction of the aerial parts of *D. nipponica*. Structures are shown in Figure 10.

### 4.7. Other Constituents

**Figure 11:**
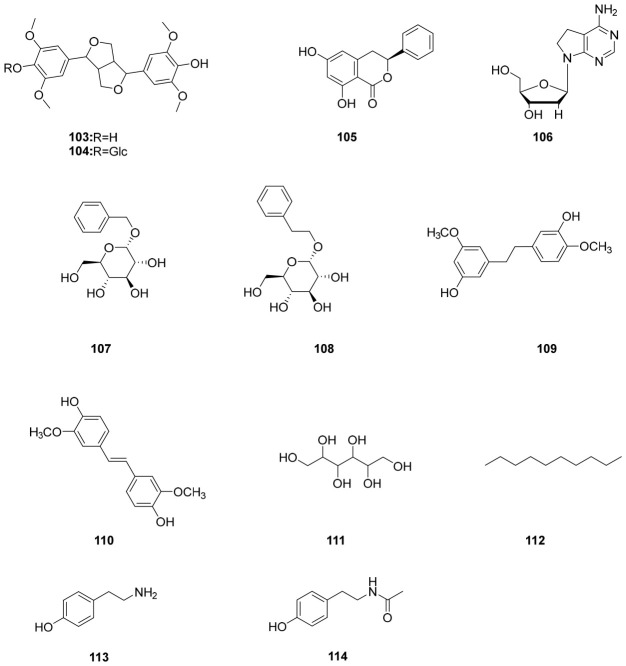
Chemical structures of other compounds in *D. nipponica*.

Liu et al. [32] isolated (+)-Syringaresinol from the 70% ethanol extract of *D. nipponica*. Zhang et al. [25] separated (+)-Syringaresinol-O-β-*D*-glucopyranoside and embran from the water-soluble part of *D. nipponica*. Lu et al. [5] isolated 3-Phenyl-6,8-dihydroxy-3,4-dihydroisocoumarin, benzyl-1-O-β-*D*-glucopyranoside, phenylethyl-1-O-β-*D*-glucopyranoside, 3’,5-dihydroxy-3,4’-methoxybibenzyl, 4,4’-dihydroxy-3,3-dimethoxy-trans-1,2-stilbene, *D*-mannitol, and *n*-decane. Structures are shown in Figure 11. A detailed summary of all chemical constituents isolated from D. nipponica is presented in Table 2.

Chemically, *D. nipponica* is characterized by a relatively high content of steroidal saponins, reaching up to 12% of the dry rhizome weight [17]. Among these, dioscin, gracillin, and methyl protogracillin are considered the principal marker compounds [8]. These constituents represent important chemotaxonomic and pharmacologically active components that differentiate *D. nipponica* from closely related species, such as *D. panthaica* and *D. septemloba*.

## 5. Pharmacological Activities

**Figure 12:**
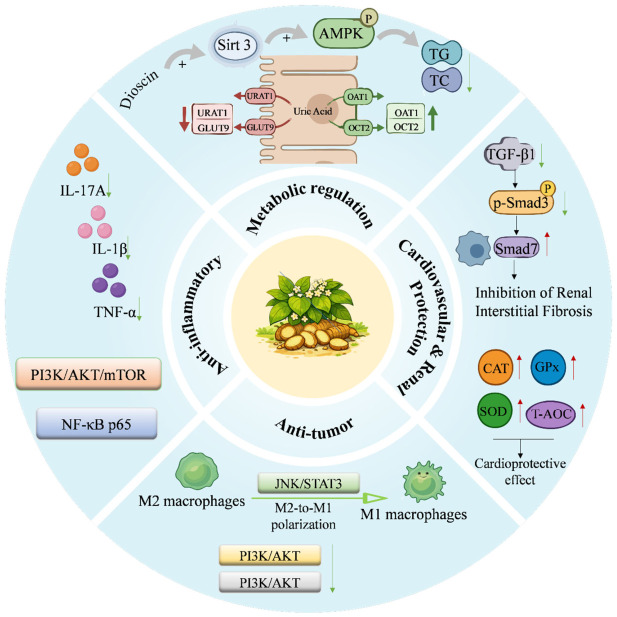
Overview of the pharmacological activities and underlying mechanisms of *D. nipponica*. The plant exhibits multiple biological effects, including anti-inflammatory, metabolic regulatory, cardiovascular and renoprotective, and anti-tumor activities. These effects are mediated through the modulation of key signaling pathways such as PI3K/Akt/mTOR, NF-κB, AMPK, and JNK/STAT3, as well as the regulation of cytokines (e.g., IL-1β, IL-17A, TNF-α), oxidative stress markers (SOD, CAT, GPx, T-AOC), and fibrosis-related factors (TGF-β1/Smad signaling). Upward and downward arrows indicate upregulation and downregulation, respectively.

The rhizome of *D. nipponica* primarily contains steroidal saponins as its major bioactive constituents. These compounds have been reported to exert a broad range of pharmacological activities, including anti-inflammatory and analgesic effects, cardiovascular and renoprotective activities, antineoplastic potential, relief of cough and asthma symptoms [1], and metabolic regulatory effects [8]. The practical and clinical translational value of *D. nipponica* steroidal saponins is deeply rooted in their structural diversity and biotransformation pathways. Spirostanol saponins (e.g., dioscin) and furostanol saponins (e.g., protodioscin) exhibit distinct pharmacokinetic behaviors; during oral administration, furostanol saponins are efficiently converted by intestinal microbiota into spirostanol forms, which possess superior membrane permeability and enhanced anti-inflammatory or anti-tumor potencies [55]. Industrially, the aglycone diosgenin serves as an irreplaceable and globally critical chemical precursor for the semi-synthesis of commercial corticosteroid hormones (such as dexamethasone and prednisone) and oral contraceptives [56,57]. This high-yield extraction value positions *D. nipponica* as a pillar resource in the modern pharmaceutical supply chain, bridging traditional ethnomedicine with large-scale industrial drug manufacturing. The comprehensive pharmacological activities and related mechanisms of *D. nipponica* are summarized in Figure 12.

### 5.1. Anti-Inflammatory and Analgesic Activity

Multiple studies to date have demonstrated that compounds isolated from *D. nipponica* exhibit significant anti-inflammatory and analgesic activities, including alleviating rheumatoid arthritis (RA), gouty arthritis (GA), and neuroinflammation, and other related inflammatory conditions [1].

RA is a chronic, systemic autoimmune disease characterized by inflammatory cell infiltration and erosion, leading to polyarticular synovitis and extra-articular manifestations [58].

Wu et al. [58] investigated the effects of dioscin, a representative steroidal saponin derived from *D. nipponica*, on collagen-induced arthritis in mice. Their findings demonstrated that dioscin significantly alleviated the severity of CIA by inhibiting Th17 cell-mediated immune responses without markedly affecting Th1 or Treg cells. In vitro assays further confirmed that dioscin suppressed the differentiation of naïve CD4^+^ T cells into Th17 cells and reduced the expression of Th17-related cytokines such as IL-17A. These results suggest that dioscin exerts its anti-arthritic effects primarily through the modulation of Th17 cell pathways, thereby providing a promising immunomodulatory strategy for RA management.

Zhai et al. [59] systematically elucidated the molecular mechanism by which TSDN (Total Saponins from *Dioscorea nipponica*) inhibits synovial angiogenesis, using the RSC-364 rat synovial cell line stimulated with a combination of TNF-α and IL-17 as an in vitro model. The results indicated that TSDN exhibited no cytotoxicity toward RSC-364 cells at concentrations ranging from 5% to 30%, while concentrations above 40% induced significant toxicity. TSDN markedly suppressed TNF-α+IL-17-induced VEGF secretion, and this inhibitory effect was positively correlated with the nuclear translocation of NF-κB/p65 protein. Moreover, the classical NF-κB inhibitor PDTC significantly reduced VEGF production, confirming the critical role of this signaling pathway. Immunohistochemical analysis further demonstrated that TSDN dose-dependently inhibited NF-κB/p65 expression, with the medium dose (20%) showing the most pronounced efficacy. Collectively, these findings suggest that TSDN exerts anti-rheumatoid arthritis effects by inhibiting NF-κB signaling, thereby downregulating VEGF expression and suppressing pannus formation in the synovium.

GA is a type of rheumatic lesion that is caused by the deposition of uric acid. It develops into tophi, nephrolithiasis, and urate nephropathy [60].

TSDN exerts dual effects of reducing uric acid levels and alleviating inflammation in a monosodium urate (MSU)-induced GA rat model [61]. The underlying mechanism may involve the regulation of the PI3K/AKT/mTOR signaling pathway, which significantly inhibits the formation of neutrophil extracellular traps (NETs) and exerts marked anti-inflammatory effects. Specifically, TSDN downregulates the mRNA and protein expression levels of AMPK and mTOR and reduces the mRNA expression of AKT and PTEN while increasing the protein expression levels of p-PI3K, p-AKT, and p-AMPK. Moreover, TSDN significantly decreases the expression levels of several key proteins associated with NET formation, such as neutrophil elastase (NE), proteinase 3 (PR3), cathepsin G (CTSG), lactoferrin (LTF), and myeloperoxidase (MPO), as well as the number of citrullinated histone 3 (CitH3)-positive cells, indicating its ability to effectively suppress NET formation. Additionally, TSDN significantly reduces the levels of pro-inflammatory cytokines IL-1β and TNF-α in the serum, further confirming its anti-inflammatory effects.

Woo et al. [33] isolated multiple diarylheptanoids from the rhizomes of *D. nipponica* and conducted in vitro tests for anti-neuroinflammatory activity. The results demonstrated that certain diarylheptanoids exhibited significant anti-inflammatory effects in the BV-2 cell model induced by lipopolysaccharide (LPS), without cytotoxicity. These compounds are likely to exert their anti-neuroinflammatory effects by inhibiting the production of nitric oxide (NO). This finding holds significant implications for the treatment of neurodegenerative diseases associated with neuroinflammation.

Zhou et al. [62] established a monosodium urate crystal-induced rat model of GA and demonstrated that TSDN exerts significant anti-inflammatory effects through multiple mechanisms. Specifically, these saponins decreased lysosomal enzyme activity and limited its extracellular release, thereby reducing tissue injury and the amplification of inflammatory responses. They also alleviated oxidative stress-induced joint damage by enhancing endogenous antioxidant enzyme activities and decreasing lipid peroxidation products, thus improving systemic redox balance. Furthermore, they modulated inflammasome activation by up-regulating the expression of components of the NLRP3 (formerly NALP3) inflammasome, thereby suppressing excessive inflammatory mediator release, correcting cytokine imbalance and immune dysfunction, and ultimately producing pronounced anti-inflammatory effects.

Tang et al. [63] found that the aqueous decoction of *D. nipponica* significantly prolonged the pain latency in mice subjected to thermal nociception tests, including extended tail-withdrawal latency upon exposure to hot water. It also markedly reduced the number of writhing responses induced by acetic acid and increased the pain threshold, as evidenced by delayed hind paw licking or shaking in the hot plate test. These results indicate that *D. nipponica* possesses notable analgesic activity.

Synthetic comparison of the above anti-inflammatory and analgesic studies reveals prominent inter-study similarities, disparities and methodological limitations that have rarely been systematically summarized in previous reviews. In terms of active components, steroidal saponins (dioscin and TSDN) are the most extensively investigated anti-inflammatory substances, acting via NF-κB, NLRP3 inflammasome and PI3K/Akt/mTOR axes to relieve joint inflammation in both RA and GA models [59,62,64]. In contrast, diarylheptanoids only exhibit anti-neuroinflammatory capacity in LPS-stimulated BV2 microglia in vitro, with no supporting in vivo animal or disease model evidence, which means their systemic anti-inflammatory potential remains underexplored. Mechanistically, dioscin targets Th17 immune differentiation for rheumatoid relief [58], whereas TSDN mainly inhibits synovial angiogenesis and neutrophil extracellular trap formation in gout models [59,64], showing distinct target preferences between monomeric saponin and total crude saponin extracts. However, obvious inconsistencies exist across experimental designs: Zhai et al. reported obvious cytotoxicity of TSDN at concentrations over 40% in synovial cells [59], while Zhou et al. achieved robust anti-inflammatory efficacy with high-dose TSDN in intact rats without describing severe systemic toxicity, which may be attributed to the different exposure systems (in vitro single cell vs. in vivo integrated organism) and variable extraction purity of saponin raw materials. Moreover, current research largely ignores synergistic interactions between saponins and diarylheptanoids, and few studies adopt unified quantitative indicators to compare the anti-inflammatory potency of different constituents under identical experimental conditions.

From a translational perspective, multiple practical barriers restrict the direct extrapolation of these preclinical anti-inflammatory data to human RA and gout clinical practice, including dosage mismatch, inadequate pharmacokinetic evidence and interspecies hepatotoxic risks as discussed in Section 7. First, effective in vivo doses of TSDN applied in rat arthritis models far exceed the daily total saponin dose (60 mg) of commercial Chuan-long-tong-mai capsules for human rheumatoid treatment. Such a huge dosage gap makes it difficult to replicate the robust joint-protective effects observed in rodents under safe clinical administration doses. Second, nearly all existing anti-inflammatory mechanism experiments rely on rodent animal models or immortalized cell lines; human primary synoviocytes, microglia or humanized disease models are rarely adopted, and human oral absorption, plasma exposure and metabolic transformation profiles of dioscin and TSDN have not been characterized systematically. Third, although short-term rodent anti-inflammatory assays do not present severe liver damage, human hepatocyte experiments and clinical long-term medication records verify that long-term high intake of steroidal saponins can induce oxidative liver injury via disrupted bile transport and cytochrome P450 metabolism [65]. Rodents possess stronger intrinsic tolerance to saponin-mediated hepatic toxicity than humans, so the safety window observed in mouse and rat anti-inflammatory studies cannot be directly applied to long-term clinical anti-rheumatic therapy. In addition, only the crude aqueous decoction of *D. nipponica* has been verified for analgesic activity in mouse nociception models [63], while the pain-relieving contribution of purified single saponin monomers remains unclear, which hinders the precise optimization of modern standardized anti-inflammatory preparations targeting inflammatory arthritis.

### 5.2. Cardioprotective Activity

Modern pharmacological studies have further confirmed that TSDN exerts significant cardioprotective effects via antioxidant mechanisms. Tang et al. [66] systematically compared the cardioprotective efficacy of total saponins from three species—*D. nipponica*, *D. panthaica*, and *D. zingiberensis*—in a rat model of acute myocardial ischemia induced by isoproterenol. All three saponin preparations significantly reduced serum levels of creatine kinase (CK), lactate dehydrogenase (LDH), and aspartate aminotransferase (AST) (*p* < 0.01), while concurrently enhancing the activity of antioxidant enzymes, including superoxide dismutase (SOD), catalase (CAT), and glutathione peroxidase (GPx), as well as increasing total antioxidant capacity (T-AOC) and inhibiting the formation of malondialdehyde (MDA), a marker of lipid peroxidation. Histological analysis using hematoxylin and eosin (H&E) staining of myocardial tissues revealed that all three total saponins markedly alleviated myocardial cell degeneration, necrosis, and inflammatory infiltration, with no significant differences in efficacy observed among them.

The activation of the mitochondrial apoptosis pathway and the release of pro-apoptotic proteins from mitochondria into the cytoplasm are controlled and regulated by the B-cell lymphoma-2 (Bcl-2) protein family, and cardiac-specific overexpression of the apoptosis inhibitor Bcl-2 can significantly reduce the size of infarcts after I/R injury [67]. Qin et al. used H9c2 cells subjected to ischemia/reperfusion (I/R) as a model to demonstrate that dioscin can reduce the expression of Bcl2-Associated X (Bax) and increase the expression of Bcl-2, thereby inhibiting the dissipation of mitochondrial membrane potential (ΔΨm) induced by I/R in H9c2 cells [67]. This indicates that dioscin can regulate mitochondrial function, prevent apoptosis and protect the heart from I/R damage.

Comparative analysis of available cardioprotective studies reveals obvious research imbalances and methodological limitations. In terms of active substances, TSDN exhibits equivalent myocardial antioxidant activity to total saponins from other Dioscorea species in rat acute ischemia models [66], yet only dioscin and diosgenin have been validated to alleviate I/R injury via regulating mitochondrial apoptosis in H9c2 cells [67]; the cardiac effects of other major steroidal saponins remain largely unexplored. Methodologically, current evidence relies narrowly on acute injury cell models and chemically induced rodent platforms [66,67]. These simplified systems lack verification in chronic, spontaneous cardiovascular disease networks, and the potential synergistic or antagonistic interactions among multiple co-existing saponins have not been clarified.

Multiple translational bottlenecks further hinder the clinical extrapolation of these preclinical datasets. First, localized human pharmacokinetic and pharmacodynamic data for TSDN and dioscin, including precise myocardial accumulation and coronary tissue exposure profiles, remain completely absent. Second, the therapeutic–toxic balance severely restricts long-term clinical administration. As detailed in Section 7, humans exhibit a lower tolerance threshold to saponin-induced systemic load and oxidative liver injury than rodents [1]. Because clinical cardiovascular therapies typically require prolonged management, this narrow safety window, which cannot be fully predicted by short-term rodent cardiac assays, strictly limits the safe clinical dosage of these saponins. Third, although crude preparations of *D. nipponica* possess a history of cardiovascular use, robust clinical trials or humanized disease models evaluating purified single constituents against ischemia/reperfusion injury are still lacking, hindering the precise optimization of modernized target-specific cardiological medications.

### 5.3. Renoprotective Activity

Chronic kidney disease (CKD) is defined as the presence of structural or functional abnormalities in the kidney for more than 3 months or a decrease in glomerular filtration rate (GFR < 60 mL/min) of unknown origin for more than 3 months [8]. Renal fibrosis, particularly tubulointerstitial fibrosis, is the common final outcome of almost all progressive chronic kidney diseases [68].

Qiao et al. [69] used a rat model of severe renal fibrosis induced by a 10% fructose solution and showed that dioscin significantly reduced serum creatinine (Cr) and blood urea nitrogen (BUN) levels. It also markedly reversed histopathological changes in renal tissue, including swelling of renal tubular epithelial cells, vacuolar degeneration, disappearance of brush borders, and coagulative necrosis. Additionally, dioscin significantly decreased the protein levels of transforming growth factor-β1 (TGF-β1) and the phosphorylation levels of Smad3 while simultaneously increasing the expression levels of Smad7, thereby exerting an inhibitory effect on renal fibrosis.

Zhong et al. [70] reported that diosgenin markedly induces autophagy via the LKB1–AMPK–mTOR signaling pathway. Meanwhile, it suppresses mitochondrial translocation of DRP1 and the expression of FIS1, both of which are associated with excessive mitochondrial fission, while up-regulating the levels of the mitochondrial fusion proteins MFN1 and MFN2, thereby ameliorating mitochondrial dynamics imbalance. On this basis, diosgenin effectively reduces the activation of caspase-3, caspase-9, and Apaf-1, attenuates mitochondrial apoptosis and renal histopathological damage, and ultimately exerts a protective effect against 3-MCPD-induced kidney injury.

Wang et al. [71] established a rat model of acute kidney injury (AKI) induced by cisplatin and found that cisplatin treatment downregulated the expression levels of Nrf2 and HO-1. Dioscin, however, significantly upregulated their expression and facilitated the nuclear translocation of Nrf2. When Nrf2 expression was inhibited, the antioxidant, anti-apoptotic, and anti-ferroptotic effects of dioscin were markedly attenuated. Additionally, dioscin ameliorated pathological changes caused by cisplatin, including degeneration and desquamation of renal tubular epithelial cells, abnormal nuclei, inflammatory cell infiltration, and mitochondrial damage. In summary, dioscin exerts renoprotection by alleviating cisplatin-induced oxidative stress, apoptosis, and ferroptosis through the activation of the Nrf2/HO-1 signaling pathway.

Li et al. [72] utilized L-02 (human hepatocytes) and NRK-52E (rat renal tubular epithelial cells) cell lines to evaluate the protective effects of dioscin against MTX-induced cellular damage. Dioscin significantly mitigated MTX-induced hepatorenal injury by modulating miR-145-5p/Sirt5-mediated oxidative stress. The study also highlighted that dioscin upregulated the expression of Sirt5, which subsequently regulated the expression of genes and proteins such as Nrf2, HO-1, SOD1, Keap1, Gst, GCLC, and NQO1 to alleviate oxidative stress, thereby exerting its renoprotective effects.

A comparison of existing renoprotective studies demonstrates a pronounced bias in screened chemical entities. Current nephroprotective research overwhelmingly centers on steroidal saponins, including dioscin and diosgenin, while the potential renal benefits of other major secondary metabolites such as phenanthrenes and diarylheptanoids remain entirely uninvestigated [69,70,71,72]. Previous trials adopt fragmented animal models covering fructose-triggered renal fibrosis, cisplatin-induced acute kidney injury and 3-MCPD-mediated nephropathy, without standardized experimental platforms for quantitative potency comparison between the two active molecules. Moreover, their protective effects have not been validated in complicated autoimmune nephropathy or spontaneous clinical CKD models, and few studies elaborate on synergistic or antagonistic interactions among different saponin components.

Multiple obstacles impede the translational progress of these preclinical findings. For one thing, human pharmacokinetic parameters of dioscin and diosgenin related to renal disposition are undocumented, and no research tracks their accumulation in kidney tissue following oral intake. For another, the interspecies disparity in hepatic susceptibility illustrated in the toxicology chapter poses hidden risks during prolonged medication, meaning that nephroprotective outcomes observed in animal models cannot accurately predict human safety profiles. Last but not least, all supportive evidence originates from cellular and rodent assays; high-quality clinical trials verifying their therapeutic efficacy in human AKI and CKD patients are still absent.

### 5.4. Anti-Tumor Activity

A number of reports have established the anti-tumor effects of *D. nipponica*, dioscin and diosgenin on various tumor cells, and the anti-tumor mechanisms were discussed, respectively [1].

Tumor-associated macrophages (TAMs) are critical immune cells in the tumor microenvironment that are classified into M1 and M2 types. M1-type TAMs exhibit anti-tumor properties, whereas M2-type TAMs display immunosuppressive and pro-tumorigenic functions. Studies have shown that TAMs predominantly exhibit M2-type macrophage functions, which are closely associated with angiogenesis and tumor progression [73]. Cui et al. [74] found that dioscin could induce the polarization of macrophages from the M2 type to the M1 type, increasing the expression of M1 markers such as NOS2 and IL-6 while decreasing the expression of M2 markers such as CD206, CD209, and Arg-1. In a subcutaneous lung cancer model, dioscin significantly reduced the proportion of M2-type TAMs in peripheral blood mononuclear cells (PBMCs), splenocytes, and tumor tissues. Overall, dioscin inhibits the progression and metastasis of lung cancer by suppressing M2 macrophage polarization and modulating the JNK and signal transducer and activator of transcription 3 (STAT3) signaling pathways, thereby affecting the immune response in the tumor microenvironment.

Lee et al. [75] reported that extracts of *D. nipponica* dose-dependently inhibited the migration and invasion of TPA-induced human cervical cancer cells (HeLa cells and SiHa cells). The underlying mechanism involves suppression of the phosphorylation of the signaling pathway, leading to the down-regulation of the expression and activation of MMP-9, thereby attenuating the metastatic potential of these cancer cells.

Dioscin has been found to inhibit the proliferation of tumor cells [76]. In endometrial cancer, dioscin upregulates p16, p21, and p27, downregulates Cyclin A/D/E and CDK2/4/6, and blocks the G0/G1 phase [77]. In osteosarcoma, dioscin significantly inhibits the proliferation of osteosarcoma cells and induces cell cycle arrest and apoptosis, with the potential mechanism being the suppression of the Akt/GSK3/β-catenin signaling pathway, thereby inhibiting the stemness properties and tumor growth of osteosarcoma [78]. Additionally, studies have shown that dioscin can inhibit the migration of tumor cells [76]. In lung cancer A549 cells, dioscin inhibits the expression of E-cadherin and N-cadherin induced by TGF-β1, as well as Snail, thus suppressing cell migration and invasion [79]. In colon cancer cells HCT116 and SW620, dioscin inhibits tumor growth, migration, and invasion by suppressing the phosphorylation of PI3K/AKT and downregulating MMP-2 and MMP-9 [80].

A critical comparison of the anti-tumor literature exposes three major research deficits hampering clinical translation. First, studies overwhelmingly focus on dioscin, whereas diosgenin, crude extracts, phenanthrenes and diarylheptanoids are rarely evaluated, with inter-component synergies uncharacterized. Second, scattered data across diverse cancer models lack unified platforms to quantitatively compare dioscin’s anti-tumor potency across tumor types. Third, most experiments only adopt tumor cell lines or subcutaneous xenografts, without verification in clinically relevant orthotopic, metastatic or PDX models. Future research should prioritize holistic multi-component evaluation, standardized tumor screening systems and high-fidelity animal models to narrow preclinical–clinical gaps.

### 5.5. Respiratory Disease Improvement Activity

Asthma is a chronic airway inflammatory disease involving various inflammatory cells and cytokines, including Th17 cells and their secreted IL-17A. IL-17A plays a significant role in airway inflammation, airway hyperreactivity, and airway remodeling in asthma [81]. Ye et al. constructed an asthma mouse model using OVA injection and aerosol sensitization. Experimental mice were randomly divided into a blank control group, a model control group, high-, medium-, and low-dose groups of TSDN, a positive control group (prednisolone acetate group), and a TSDN + prednisolone acetate group. From day 18 of the experiment, mice in each group were given different doses of TSDN or prednisolone acetate until day 55. The results showed that TSDN could significantly inhibit the proliferation of the airway wall and bronchial smooth muscle in chronic persistent asthma mice and suppress the expression of IL-17A in bronchoalveolar lavage fluid and lung tissue homogenate in a dose-dependent manner [82].

Wang et al. [83] conducted experiments using human airway smooth muscle cells (HASMCs), exposing the cells to varying concentrations of IL-17A before treating them with different concentrations of TSDN. The results showed that IL-17A increased the stiffness and traction force of HASMCs in a dose- and time-dependent manner, while TSDN could dose-dependently mitigate these changes. Additionally, TSDN significantly regulated IL-17A-induced proliferation, migration, and cytoskeletal remodeling of HASMCs. The potential mechanism may involve the inhibition of IL-17A receptors.

Yang et al. [84] investigated the anti-inflammatory effects of diosgenin on asthma and its underlying molecular mechanisms. Through in vitro and in vivo experiments, they found that diosgenin significantly reduced the infiltration of inflammatory cells and the secretion of pro-inflammatory cytokines (such as TNF-α, IL-1β, and IL-6). This effect may be achieved by enhancing the expression and activity of glucocorticoid receptors (GRs) and inhibiting the expression of heat shock protein 70 (HSP70). Additionally, diosgenin upregulated the expression of anti-inflammatory genes (such as SLPI, TTP, GILZ, and MKP-1) and downregulated NF-κB expression, further reducing the production of pro-inflammatory cytokines. These findings suggest that diosgenin may exert its anti-inflammatory effects by activating GR-mediated anti-inflammatory signaling pathways. This study provides evidence for the clinical application of diosgenin as a natural anti-inflammatory agent, especially in the treatment of inflammatory diseases related to GRs, such as asthma.

A cross-review of anti-asthma research highlights differentiated targeting patterns between the crude saponin mixture and the single monomer, alongside neglected research directions unique to respiratory disorders. TSDN primarily suppresses the IL-17A signal cascade to reverse pathological bronchial smooth muscle remodeling, whereas diosgenin exerts anti-inflammatory effects by amplifying glucocorticoid receptor-mediated anti-inflammatory programs, representing two distinct intervention strategies for asthmatic inflammation [82,83,84]. Furthermore, all current asthma assays focus solely on inflammatory cytokines and tissue remodeling markers; no functional readouts such as bronchial spasm relief or pulmonary ventilation indices are reported to evaluate direct bronchodilatory potency. Apart from saponins, few studies have explored the airway-protective potential of aerial-derived non-saponin secondary metabolites such as phenanthrenes and diarylheptanoids, despite their proven anti-inflammatory activity in other disease models.

From a translational perspective, respiratory therapy faces unique barriers not observed in other disease fields. Asthma requires sustained local drug exposure in lung tissue, yet there is no research clarifying the pulmonary tissue distribution of oral saponins or assessing the feasibility of inhalation administration. Moreover, long-term asthma management demands repeated daily medication; combined with interspecies hepatic susceptibility gaps described in the toxicology section, continuous oral intake raises cumulative liver damage risks that cannot be predicted via short-term animal assays. At present, no controlled clinical trials have confirmed whether saponin preparations can reduce reliance on inhaled hormones in asthmatic patients.

### 5.6. Regulating Metabolic Activity

Common diseases caused by abnormal lipid metabolism include obesity [85], hyperlipidemia [86], nonalcoholic fatty liver disease (NAFLD) [87], and atherosclerosis [88]. Dioscin, a natural product, has been shown to have a good lipid-lowering effect [8]. Dioscin can regulate the Sirt1/AMP-activated protein kinase (AMPK) signaling pathway and the liver X receptor (LXR) signaling pathway and inhibit sterol regulatory element-binding proteins (SREBPs) and their downstream lipid metabolism-related proteins, thereby affecting plasma cholesterol, triglyceride, and fatty acid levels [89].

Liu et al. [90] utilized HFD-induced C57BL/6J mice and ob/ob mice as experimental models to evaluate the effects of dioscin by analyzing serum and hepatic biochemical parameters, mRNA, and protein expression levels. The study demonstrated that dioscin significantly reduced serum levels of alanine aminotransferase (ALT), aspartate aminotransferase (AST), insulin, free fatty acids (FFA), total cholesterol (TC), and triglycerides (TG). Meanwhile, it increased the hepatic levels of superoxide dismutase (SOD) and decreased MDA levels. In addition, dioscin upregulated the expression of ACADM, PPARα, ACADS, ACSL1, and ACSL5, and downregulated LXRα, thereby regulating fatty acid synthesis and metabolism and reducing hepatic lipid accumulation. It also downregulated HMGCR, HMGCS1, and SREBP-2, and upregulated GPAT to regulate TG and TC synthesis, thereby improving hepatic lipid metabolism.

Wang et al. [91] utilized male Sprague-Dawley (SD) rats to induce hyperlipidemia through a high-fat diet containing 5% cholesterol, 0.5% bile salt, and 0.2% thioacetate. The SD rats were randomly divided into groups and treated with trillin (0.5 mg/kg), lovastatin (10 mg/kg), or solvent (0.5% DMSO) once daily from day 14 to day 28. The anti-hyperlipidemic and antioxidant effects of trillin were assessed by measuring the levels of cholesterol, triglycerides, LDL, and HDL in the blood, as well as lipid peroxidation and SOD activity. The results showed that trillin significantly reduced the levels of cholesterol and triglycerides in the blood of hyperlipidemic rats, with a reduction of about 90% in cholesterol levels and a significant decrease in triglyceride levels. Additionally, trillin lowered LDL levels and partially reduced HDL levels.

Uric acid, a substance with poor solubility, is an end product of purine metabolism in humans. Hyperuricemia, defined as high levels of blood uric acid, is recognized as a risk factor for gout, cardiovascular disease, hypertension, diabetes, and chronic kidney disease [92]. The excretion of uric acid primarily occurs through the kidneys and intestines, with the kidneys accounting for more than 70%. The reabsorption of uric acid in the kidneys is mainly mediated by the urate transporter 1 (URAT1) and the glucose transporter 9 (GLUT9) [93].

Zhang et al. [94] used SD rats and Kunming mice and induced hyperuricemia by administering a uricase inhibitor (PO) and a uric acid production promoter (adenine). Dioscin was orally administered at different doses (25 mg/kg and 50 mg/kg) for 14 to 28 days. The results showed that in the hyperuricemia rat model, dioscin significantly reduced serum uric acid levels and the area under the curve (AUC). In the adenine and PO-induced hyperuricemia mouse model, dioscin significantly decreased serum uric acid and creatinine levels, increased uric acid and creatinine clearance, and raised the fractional excretion rate of uric acid. In the kidneys of dioscin-treated mice, the expression of GLUT9 was significantly downregulated, while the expression of OAT1 was upregulated. Additionally, Zhang et al. used a human renal epithelial cell line stably expressing hURAT1 (HEK293-hURAT1) to assess the inhibitory effects of dioscin metabolites on uric acid reabsorption and HCT116 cells to evaluate the transcellular transport of uric acid in the intestine. The results indicated that Diosgenin and its metabolite Tigogenin significantly increased the transcellular transport of uric acid in HCT116 cells, and this effect was at least partially mediated by ABCG2.

Su et al. [95] used male Kunming mice and induced hyperuricemia by administering a uricase inhibitor (potassium oxonate, PO, 250 mg/kg). Dioscin was orally administered at different doses (319.22, 638.43, and 1276.86 mg/kg/day) for 10 days. Uric acid and creatinine levels in serum and urine were determined by HPLC and HPLC-MS/MS, respectively. Xanthine oxidase (XO) activity in mouse liver was examined in vitro. Protein levels of organic anion transporter 1 (mOAT1), urate transporter 1 (mURAT1), and organic cation transporter 2 (mOCT2) in the kidneys were analyzed by Western blotting. The results indicated that dioscin treatment significantly reduced the increase in serum uric acid and creatinine levels caused by PO. The mechanism involved the regulation of the expression of mOAT1, mURAT1 and mOCT2.

Comparative analysis of metabolic regulatory research reveals two distinct research branches: lipid modulation and urate metabolism regulation, each with characteristic strengths and understudied directions. For lipid disorders, dioscin governs multi-lipid metabolic axes, including Sirt1/AMPK and LXR pathways, to mitigate hepatic steatosis in obese rodent models [90], while trillin exhibits potent cholesterol-lowering activity in hyperlipidemic rats via antioxidant modulation [91]. Existing work mainly focuses on hepatic lipid accumulation, yet few studies comparatively analyze whether these saponins exert consistent regulatory effects on peripheral adipose tissue metabolism. As for hyperuricemia, dioscin regulates renal and intestinal urate transporters (GLUT9, OAT1, ABCG2) across mouse, rat and human cell platforms [94,95], showing dual renal–intestinal urate-excreting advantages rarely observed in other bioactive components. However, current investigations pay little attention to the interactive linkage between lipid disturbance and hyperuricemia, despite their frequent coexistence in metabolic syndrome patients. Non-saponin metabolites from *D. nipponica* have also received limited exploration for combined metabolic disorder intervention.

### 5.7. Other Activities

Tan et al. validated the antidepressant effects of dioscin by constructing a chronic unpredictable mild stress (CUMS) mouse model of depression. The potential mechanism may be related to the regulation of oxidative stress and energy metabolism mediated by UCP2 [96].

Yu et al. [97] discovered that protodioscin (PD) exhibits antibacterial activity against Raoultella ornithinolytica B1645-1B, a strain producing New Delhi metallo-β-lactamase 1 (NDM-1) and resistant to carbapenems. When combined with imipenem (IMP), PD demonstrates synergistic antibacterial effects, reducing the minimum inhibitory concentration (MIC) of IMP. It also inhibits the mRNA synthesis and protein expression of the NDM-1 gene and reduces its enzyme activity, thereby reversing antibiotic resistance.

Yang et al. [98] reported that the total saponins of *D. nipponica* exert significant neuroprotective effects in rats with cerebral ischemic injury induced by the middle cerebral artery occlusion (MCAO) model. Treatment markedly reduced neurological deficit scores, decreased cerebral infarct volume, and improved the histopathological morphology of brain tissue. The underlying mechanisms are mainly associated with the attenuation of oxidative stress through increasing serum superoxide dismutase (SOD) activity and decreasing the level of MDA. In addition, the treatment down-regulated the expression of MMP-2 in brain tissue, thereby protecting the blood–brain barrier and alleviating cerebral edema.

Cai et al. [99] reported that TSDN exerts protective effects against skin photoaging. Mechanistically, TSDN directly targets and binds to STAT3, thereby suppressing its aberrant activation. This modulation subsequently regulates the downstream Nrf2/HO-1 signaling pathway associated with oxidative stress and the Bax/Bcl-2 apoptotic pathway, significantly inhibiting ultraviolet B (UVB) radiation-induced oxidative stress and apoptosis. Consequently, TSDN promotes the repair of the skin barrier, protects collagen fibers, and alleviates the pathological damage associated with photoaging.

Zheng et al. isolated the active compound Gracillin from Alpinia zerumbet and found that it exhibits significant antiparasitic activity. Compared to the control group, Gracillin significantly reduced the number of *Ichthyophthirius multifiliis* in a dose-dependent manner [100].

Comparative overview of these miscellaneous bioactivities reveals scattered, fragmented research coverage with uneven attention paid to distinct pharmacological potentials. Current scattered findings cover antidepressant, anti-drug-resistant bacterial, cerebral neuroprotective, anti-photoaging and antiparasitic effects, yet each biological function is supported by only one or two independent studies without follow-up cross-verification. Among all tested compounds, saponins dominate existing explorations, while non-saponin constituents have barely been assessed for these miscellaneous pharmacological phenotypes. Additionally, each activity branch adopts isolated disease models without unified evaluation systems, and few studies explore whether these minor bioactivities could exert auxiliary therapeutic value for the core clinical indications of *D. nipponica*, such as rheumatic disorders and metabolic syndromes.

From a translational perspective, multiple practical limitations restrain further exploration of these understudied effects. Relevant human pharmacokinetic data targeting the brain, skin and peripheral infectious tissues remain scarce. Consistent with the hepatotoxic discrepancy described in the toxicology section, long-term administration for auxiliary therapy carries latent hepatic risks that cannot be fully predicted via short-term rodent tests. In addition, nearly all relevant evidence is limited to preliminary preclinical observations; relevant clinical trials to confirm auxiliary curative effects are still lacking.

To facilitate comparison of the available pharmacological evidence, the representative medicinal properties of *D. nipponica* are summarized in Table 3. The table systematically summarizes the plant parts, extract types or representative active constituents, experimental models, dosages or concentrations, and major pharmacological findings reported in previous studies. This overview highlights the close relationship between different plant materials, phytochemical constituents, and their corresponding biological activities and provides a concise reference for future pharmacological and translational studies.

While steroidal saponins remain the primary focus, the non-saponin fractions—specifically flavonoids and phenolic acids—contribute critically to the comprehensive therapeutic profile of *D. nipponica*. The identified flavonoids, including rutin and kaempferol-3-O-rutinoside, demonstrate potent anti-adipogenic effects by inhibiting lipid accumulation in preadipocytes while simultaneously protecting microvascular endothelial cells from oxidative damage [84,102]. Concurrently, phenolic components such as gallic acid, methyl gallate, and protocatechuic acid exert robust radical-scavenging capacities [103]. Mechanistically, these phenolic acids alleviate systemic inflammation by downregulating COX-2 and suppressing the intracellular oxidative cascade [104]. The presence of these polyphenolic networks suggests that the therapeutic efficacy of *D. nipponica* extracts relies on a multi-target, multi-component synergy between saponins and co-existing polar metabolites.

## 6. Clinical Applications

### 6.1. Treatment of Arthritis

RA, osteoarthritis (OA), and other inflammatory joint disorders represent the most extensively investigated clinical indications of *D. nipponica*. Several Chinese patent medicines and pharmaceutical preparations containing *D. nipponica*, such as “Gulong Jiaonang”, have been used clinically for the treatment of rheumatic diseases because of their anti-inflammatory and analgesic properties [105,106]. Available clinical studies suggest that these preparations may alleviate joint pain, improve physical function, and reduce inflammatory responses when administered either alone or in combination with conventional therapies [1]. These therapeutic effects are considered to be closely associated with the anti-inflammatory and immunomodulatory activities of steroidal saponins, particularly dioscin and related constituents.

### 6.2. Treatment of Chronic Brucellosis

*D. nipponica* has also been investigated as an adjunctive therapy for chronic brucellosis. An early clinical study reported that *D. nipponica* preparations improved clinical symptoms in patients with chronic brucellosis and were generally well tolerated [107]. Subsequently, Liu et al. [108] further evaluated a modified Sini San prescription supplemented with *D. nipponica* in patients with chronic brucellosis and reported improvements in clinical manifestations and laboratory indicators compared with conventional treatment alone. These findings suggest that *D. nipponica* may provide therapeutic benefits as an adjunct to standard antimicrobial therapy, possibly through its anti-inflammatory and immunomodulatory activities.

### 6.3. Treatment of Chronic Bronchial Asthma

Clinical investigations of *D. nipponica* and its preparations have provided preliminary evidence supporting its adjunctive use in chronic bronchial asthma. Zhang Yanping et al. [109] reported that a combination of *D. nipponica* with acupoint injection of ligustrazine improved clinical symptom control in asthma patients, with reductions in wheezing frequency and cough severity, alongside improvements in lung function indices such as FEV_1_ and overall effective rate compared with baseline therapy alone. Similarly, Zhao et al. [110] investigated a modified formulation of *D. nipponica* decoction combined with Dingchuan decoction and observed enhanced symptom relief and reduced recurrence tendency in patients with bronchial asthma, suggesting a potential synergistic effect in relieving airway obstruction and inflammation. In a study focusing on severe asthma, Lei et al. [111] reported that acupoint injection of *D. nipponica* was associated with rapid alleviation of dyspnea and improved short-term clinical response rates, particularly in acute symptom control settings.

### 6.4. Other Indications

Besides arthritis and chronic respiratory diseases, therapeutic applications of *D. nipponica* preparations have been extended to hyperlipidaemia, coronary heart disease, chronic pelvic inflammatory disease and fracture rehabilitation [1,8,112]. Cardiovascular research is the most mature branch among these indications, with “Di’ao Xinxuekang” being a classic marketed herbal formulation clinically indicated for lipid disorders and coronary heart disease, whose lipid-regulating and myocardial protective effects are supported by multiple clinical observational studies [113]. To systematically summarize the clinical therapeutic spectrum of *D. nipponica* described above, the major clinical indications, representative preparations, therapeutic effects, and underlying mechanisms are organized in Table 4.

## 7. Toxicology Studies

### 7.1. Safety Evaluation

Acute and sub-chronic tests show that total saponins of *D. nipponica* are generally well tolerated. In mice, the oral LD_50_ is 1.85 g/kg and the intraperitoneal LD_50_ is 0.72 g/kg; clinical signs were transient ataxia and reduced locomotion, resolving within 24 h [117]. A 28-day gavage study in rats at 50, 150 and 450 mg/kg/day revealed no mortality, but the high dose elevated ALT (≈2-fold) and induced mild centrilobular hydropic change; both effects reversed after a 2-week recovery period. Dioscin, the major saponin, generated reactive oxygen species in a HepG2 cell model, yet cytotoxicity (IC_50_ 35 µM) was attenuated by co-incubation with N-acetylcysteine, indicating an oxidative mechanism that is saturable and dose-dependent [118]. No genotoxicity was detected in the Ames or mouse micronucleus assays at ≤5000 µg/plate or 2000 mg/kg, respectively. Overall, doses ≤ 100 mg total saponins/day in adults are considered safe; monitoring of hepatic enzymes is advised for long-term or high-dose regimens [1].

Steroidal saponins, particularly dioscin, gracillin, protodioscin, and diosgenin, are recognized as the major bioactive constituents of *D. nipponica* [18]. Current toxicological evidence is mainly derived from studies of total steroidal saponins and dioscin [72,80,119], whereas the safety profiles of other major constituents remain insufficiently investigated. Available toxicological studies suggest that total steroidal saponins from *D. nipponica* are generally well tolerated at therapeutic doses [1]. However, experimental studies indicate that excessive exposure to dioscin may induce oxidative stress and hepatocellular damage [65], highlighting the importance of dose optimization during clinical application. These findings indicate a relatively wide therapeutic window but also highlight the importance of dose optimization and long-term safety evaluation.

### 7.2. Toxicological Mechanisms of Major Steroidal Saponins

Although *D. nipponica* is generally considered safe at therapeutic doses, increasing evidence suggests that several major steroidal saponins may induce cytotoxicity under high-dose or prolonged exposure. Current studies indicate that oxidative stress, mitochondrial dysfunction, apoptosis, and inflammatory responses are the principal mechanisms underlying the toxicological effects of these constituents [1]. Among them, dioscin has been the most extensively investigated, whereas toxicological evidence for gracillin, protodioscin, and pseudoprotodioscin remains relatively limited.

Zhang et al. [65] reported that high-dose dioscin exerts hepatotoxicity, and the underlying toxic mechanism may be associated with the activation of the aryl hydrocarbon receptor (AhR) and upregulation of cytochrome P450 1A (CYP1A) expression. Yang et al. reported that dioscin can induce time-dependent progressive cascade liver injury in in vivo animal experiments combined with an in vitro human normal hepatocyte L-O_2_ cell model [120]. Short-term exposure to dioscin inhibits the activities of hepatic membrane Na^+^-K^+^-ATPase and Ca^2+^-Mg^2+^-ATPase, disrupts intracellular ionic homeostasis and triggers intracellular calcium accumulation. Meanwhile, it downregulates the expression of bile acid transporters sodium taurocholate cotransporting polypeptide (NTCP) and bile salt export pump (BSEP), hinders bile acid excretion and thereby induces cholestasis. With prolonged exposure, dioscin reduces the activities of antioxidant enzymes such as SOD and glutathione peroxidase (GSH-Px), elevates the level of lipid peroxide MDA and provokes oxidative stress injury. It also upregulates the protein expression of cytochrome P450 2E1 (CYP2E1) and cytochrome P450 3A4 (CYP3A4), excessively activating the phase I metabolic pathway to produce abundant toxic metabolites and aggravate hepatic damage. Long-term continuous stimulation further disrupts mitochondrial structure, decreases the activity of the mitochondrial marker enzyme succinate dehydrogenase (SDH), induces mitochondrial permeability transition, and ultimately initiates hepatocyte apoptosis or necrosis. Overall, the toxic injury exhibits obvious time dependence, and long-term oral administration may cause irreversible liver damage. Protodioscin has also been reported to induce ROS production, endoplasmic reticulum stress, mitochondrial dysfunction, and apoptosis in several cancer cell models. However, whether these mechanisms contribute to its toxicological effects under physiological conditions remains unclear [121].

## 8. Conservation and Cultivation

### 8.1. Resource Status

Wild *D. nipponica* populations have declined >60% over the past two decades as a consequence of intensive harvesting for diosgenin manufacture and habitat loss along easily accessible hillsides [122]. Wild populations of *D. nipponica* have declined in parts of northeastern China owing to long-term overharvesting and increasing market demand. Consequently, the species has been included in provincial protected plant lists, highlighting the need for strengthened conservation and sustainable utilization [123]. In addition, environmental factors, harvest season, and habitat conditions have been shown to influence the accumulation of steroidal saponins and diosgenin in rhizomes, emphasizing the importance of standardized cultivation and resource management [124]. Consequently, conservation strategies combining in situ protection (nature reserves, harvest rotation, community co-management) and ex situ repositories (botanical gardens, seed banks, DNA barcoding) are urgently required to safeguard genetic diversity and prevent local extinction.

### 8.2. Propagation Methods

Traditional rhizome division gives 85–90% survival but only 3- to 5-fold multiplication per year and acts as a vector for Fusarium rot, while seed germination remains <20% after 12–16 weeks of cold stratification [125]. To meet commercial demand without further wild depletion, several laboratories have optimized in vitro protocols. On Murashige–Skoog medium supplemented with 2.0 mg/L 6-benzyladenine + 0.2 mg/L NAA, callus induced from mature seeds produced adventitious shoots at 90% frequency; rhizome-callus achieved 88% shoot differentiation with 3.0 mg/L 6-BA + 2.0 mg/L NAA, and rooting (86%) was obtained on 1/2-strength MS plus 0.5 mg/L NAA. Hairy-root lines generated by Agrobacterium rhizogenes R1601 accumulated 3.7% diosgenin within 5 weeks, offering a continuous bioreactor source of steroidal sapogenins. Cryopreservation of shoot tips (encapsulation-dehydration, LN) maintained 75% regrowth and genetic fidelity for long-term germplasm banking [126]. These biotechnological platforms provide the basis for certified seedling production, elite cultivar breeding and metabolite-based manufacturing that relieve harvesting pressure on natural stands.

## 9. Discussion

Although substantial progress has been made in elucidating the chemical composition and pharmacological activities of *D. nipponica*, current research remains fragmented across three largely disconnected trajectories: traditional pharmacological validation using in vitro and in vivo disease models, molecular mechanism studies centered on canonical inflammatory and metabolic pathways (e.g., NF-κB, PI3K/Akt, AMPK), and resource-oriented investigations focusing on cultivation, propagation, and biomass engineering.

However, limited efforts have been devoted to integrating these trajectories into a unified framework linking chemical classes, molecular targets, and therapeutic applications. As a result, the majority of studies remain reductionist, focusing on single compounds and isolated pathways, which constrains both mechanistic depth and translational relevance. Establishing an integrative, multi-component, and systems-level research paradigm is therefore essential for advancing the rational development of *D. nipponica*-derived therapeutics.

### 9.1. Problems Associated with the Extraction of Bioactive Compounds

Currently, the main extraction and isolation methods for active compounds in *Dioscorea nipponica* Makino remain traditional solvent extraction and conventional chromatographic techniques such as HPLC, GC-MS, and LC-MS [18,43]. Only a few studies have attempted to assist isolation using biotechnological approaches, which necessitates consideration of whether existing technologies have the issue of missing low-content active ingredients. As mentioned earlier, compounds such as phenols and flavonoids in *D. nipponica* have only been isolated in small quantities from stems, leaves, or water-soluble fractions. Although diarylheptanoids exhibit anti-neuroinflammatory activity, the number of isolated compounds of this class is far lower than that of rhizome saponins [34,52]. This may be associated with the underutilization of aboveground-part resources.

In future research, we should focus on optimizing isolation technologies to achieve full utilization of resources and reduce waste. For instance, integrating transcriptomics and metabolomics can help identify key genes regulating steroidal saponin biosynthesis in *D. nipponica*, paving the way for efficient production of bioactive components via synthetic biology.

Importantly, recognizing the practical and economic value of flavonoids and phenols opens up sustainable avenues for biomass utilization. Historically, the harvesting of *D. nipponica* has focused exclusively on the underground rhizomes, leading to the massive discarding and environmental wastage of the aerial stems and leaves. However, phytochemical profiling reveals that these aerial parts serve as a rich, renewable reservoir for kaempferol derivatives, rutin, and diverse phenolic acids. Characterized by high stability and strong antioxidant indices, these non-saponin fractions possess significant commercial potential as natural functional additives in the nutraceutical, cosmetic, and anti-aging industries. Furthermore, incorporating these distinctive phenols and flavonoids into the plant’s standardized quality-control (QC) frameworks as co-markers will markedly elevate batch-to-batch clinical reproducibility, transforming agricultural waste into high-value pharmaceutical and industrial assets.

### 9.2. Pharmacological Effects

*D. nipponica* has attracted considerable attention because of its broad spectrum of pharmacological activities. Collectively, the available studies consistently demonstrate that steroidal saponins, particularly dioscin and diosgenin, are the major contributors to the anti-inflammatory, anti-tumor, cardioprotective, renoprotective, and metabolic regulatory effects of this plant [8,60,67,76,83,127]. Despite differences in experimental models, most studies reached similar conclusions that these compounds exert therapeutic effects primarily through the regulation of inflammatory responses, oxidative stress, apoptosis, and immune function.

One notable finding across different studies is the remarkable consistency in anti-inflammatory mechanisms. Independent investigations using LPS-induced macrophages, GA, asthma, and acute inflammatory animal models consistently demonstrated that *D. nipponica* extracts or steroidal saponins suppress the production of pro-inflammatory mediators, including TNF-α, IL-1β, IL-6, and NO, mainly through the modulation of the NF-κB, NLRP3 inflammasome, MAPK, and PI3K/Akt signaling pathways [1,8,61,62,69,95]. These convergent findings suggest that regulation of inflammatory signaling represents one of the most reproducible pharmacological characteristics of *D. nipponica*.

However, some discrepancies exist among published studies. While most investigations focused almost exclusively on steroidal saponins, recent studies have demonstrated that diarylheptanoids and phenols also possess significant anti-inflammatory and immunomodulatory activities [52]. These observations indicate that the pharmacological activities of *D. nipponica* cannot be fully explained by steroidal saponins alone. Instead, different classes of phytochemicals may contribute to distinct therapeutic effects through complementary molecular targets. Nevertheless, compared with steroidal saponins, these non-saponin constituents remain considerably underexplored with respect to their chemical diversity, structure–activity relationships, pharmacokinetic properties, and molecular mechanisms.

The differences among studies may also be attributed to variations in extraction methods, phytochemical composition, experimental models, dosage regimens, and analytical techniques [17,20]. Some studies evaluated purified compounds such as dioscin or diosgenin, whereas others employed crude extracts or total saponin fractions, making direct comparisons difficult. Moreover, differences in geographical origin, cultivation conditions, and quality-control standards may further contribute to variability in chemical composition and pharmacological outcomes [128]. Therefore, establishing standardized extraction procedures, quality-control markers, and unified experimental protocols will be essential for improving the reproducibility and comparability of future studies.

Secondly, research on *D. nipponica* has mainly focused on individual steroidal saponins, whereas relatively little attention has been paid to the synergistic effects among multiple constituents. “Gulong Jiaonang”, a Chinese patent medicine composed of *D. nipponica* rhizome and canine limb bone, is clinically used for the treatment of RA and other rheumatic disorders because of its analgesic and anti-rheumatic effects [105], and no serious adverse reactions have been reported in the available literature. However, despite its long-standing clinical use, the pharmacological mechanisms, the contribution of individual constituents, and the safety profile of this preparation remain insufficiently investigated. Therefore, future studies should focus on elucidating the multi-component, multi-target mechanisms underlying its therapeutic effects and establishing systematic evidence for its clinical efficacy and safety.

Although extensive investigations have systematically validated the diverse pharmacological profiles of steroidal saponins from *D. nipponica*, including anti-inflammatory, renoprotective, anti-tumor and cardioprotective activities, toxicological research on these bioactive constituents remains severely underdeveloped and lags far behind pharmacological studies. Contradictory findings exist among available safety assessments: some acute and subchronic toxicity tests conducted in rodents report satisfactory tolerance to therapeutic doses of total saponins without evident hepatic toxicity, which may lead to a misleading conclusion that steroidal saponins exert no hepatotoxic effects. Nevertheless, mechanistic toxicological studies on high-purity monomeric saponins, together with clinical adverse reaction surveillance of *D. nipponica*-containing proprietary medicines, collectively demonstrate that these steroidal saponins possess definite hepatotoxic potential.

A systematic collation of published literature reveals that the detection of hepatic toxicity induced by steroidal saponins is strongly correlated with experimental subjects, presenting distinct interspecies disparities. It can be reasonably inferred that hepatotoxicity triggered by steroidal saponins manifests prominently in in vitro human hepatocyte models and during long-term human clinical administration scenarios, whereas rodent laboratory animals (rats and mice) exhibit inherent tolerance toward these compounds, with milder and more reversible liver lesions under equivalent dosages. Such interspecies tolerance differences substantially restrict the predictive value of rodent toxicity assays. Although animal models enable preliminary screening of general toxic risks and partial toxic signaling pathways, they cannot fully recapitulate human-specific characteristics, including drug metabolism profiles, hepatic transporter expression, bile acid excretion dynamics, and long-term clinical exposure conditions. Consequently, toxicity data solely derived from rodent experiments fail to accurately predict latent hepatic injury risks in human patients receiving long-term or high-dose medications. The current scarcity of human-relevant toxicological evidence creates a critical gap hindering the rational clinical administration and translational pharmaceutical development of *D. nipponica*.

Collectively, steroidal saponins remain the best-characterized bioactive constituents with the greatest translational potential, whereas flavonoids and phenolic compounds are emerging as promising complementary pharmacophores. Future studies should strengthen comparative investigations among these chemical classes to clarify their respective contributions and potential synergistic effects in the pharmacological activities of *D. nipponica*.

## 10. Conclusions and Future Perspectives

Over the past decade, *D. nipponica* has emerged as a clinically relevant botanical, consistently affording 2–4% rhizome steroidal saponins—dioscin, gracillin and diosgenin—that orchestrate anti-rheumatic, anti-inflammatory, anti-hyperlipidaemic, cardioprotective and bronchodilator effects via NF-κB/NLRP3, PI3K/Akt and MAPK axis modulation coupled with 5-LOX, COX-2 and HMG-CoA reductase inhibition [129]. These mechanisms have translated into measurable patient benefit in rheumatoid arthritis, chronic brucellosis, bronchial asthma and myocardial ischaemia, positioning the species as a renewable feedstock for corticosteroid semi-synthesis and a direct source of modern botanical drugs or functional ingredients. Yet a >60% decline in wild populations over two decades of over-harvesting, together with toxicological evidence indicating a threshold of reversible hepatocyte oxidative stress that caps the human saponin dose at ≈100 mg/day, underscores that future value can only be realised by merging conservation with biotechnology [118]. Genome-guided breeding toward cultivars exceeding 5% diosgenin, scaled-up in vitro propagation, cryobanking and Agrobacterium-driven bioreactors promise sustainable, high-quality biomass without further encroachment on natural stands, while GCP-compliant trials and synthetic-biology-enabled spirostanol engineering should refine dosage, pharmacokinetics and receptor selectivity and minimise toxicity. Integrating these strategies will secure *D. nipponica* as a long-term, evidence-based pharmaceutical and nutraceutical resource.

## Figures and Tables

**Table 1:** Reported contents and analytical methods of major steroidal saponins in *D. nipponica*.

Compounds	Reported Content Range	Analytical Method	Ref.
Protodioscin	1.15~4.84%	HPLC-QTOF-MS/QAMS	[17,19]
Protogracillin	0.19~1.95%	HPLC	[17,20]
Methylprotodioscin	0.32~1.32%	HPLC/LC-MS	[17,20]
Pseudoprotodioscin	0.02~0.19%	HPLC/LC-MS	[17,21]
Dioscin	0.68~3.81%	HPLC	[17,20]
Gracillin	0.06~0.52%	HPLC	[17,20]

**Table 2:** The chemical constituents isolated from *D. nipponica*.

Class	Chemical Compounds	Refs.
Spirostanol Saponins	Dioscin (**1**)	[38,39]
	Gracillin (**2**)	[40,41]
	Trillin (**3**)	[23,42]
	Dioscin Dc (**4**)	[24]
	Diosgenin-3-O-{α-*L*-rhamnopyranosyl-(1→2)-[α-*L*-rhamnopyranosyl-(1→3)]}-β-*D*-glucopyranoside (**5**)	[24]
	Diosgenin (**6**)	[43,44]
	Progenin II (**7**)	[45]
	Progenin III (**8**)	[45,46]
	Diosgenone (**9**)	[26]
	Diosgenin-3,6-dione (**10**)	[26]
	3-O-[α-*L*-rhamnopyranosyl(1→2)-{α-*L*-rhamnopyranosyl(1→4)}-(6’-O-hexadecanoyl)-β-*D*-glucopyranosyl]-25-(*R*)-spirost-5-en-3-β-ol (**11**)	[25]
	Smilagenone (**12**)	[27]
	*Zingiberenin B* (**13**)	[24]
	25-*D*-spirosta-3,5-diene (**14**)	[25]
Furostanol Saponins	Protodioscin (**15**)	[39,41]
	Protogracillin (**16**)	[39,41]
	(25*R*)-26-O-β-*D*-glucopyranosyl-furost-5(**6**)-en-3β,22α,26-triol-3-O-[α-*L*-rhamnopyranosyl-(1→4)]-β-*D*-glucopyranoside (**17**)	[32]
	Kikuba-saponin (**18**)	[28]
	3β,26-dihydroxy-25(*R*)-furosta-5,20(**22**)-dien-26-O-β-*D*-glucopyranoside (**19**)	[29]
	Pseudoprotodioscin (**20**)	[47,48]
	Methylprotodioscin (**21**)	[49]
	Methylprotogracillin (**22**)	[17]
	Diosnipponicoside A–O (23–37)	[32]
	25*R*-Dracaenoside O (**38**)	[18]
	25*R*-Dracaenoside P (**39**)	[32]
	hypoglaucin G (**40**)	[32]
	anguivioside XV (**41**)	[32]
	3-O-[α-*L*-rhamnopyranosyl-(1→2)]-[β-*D*-glucopyranosyl-(1→3)]-β-*D*-glucopyranosyl-26-O-β-*D*-glucopyranosyl-cholest-5-en-16,22-dione	[32]
Diarylheptanoids	1,7-bis(4-Hydroxyphenyl)-1,4,6-heptatrien-3-one (**43**)	[6]
	1,7-bis(4-Hydroxyphenyl)-4,6-heptadien-3-ol (**44**)	[6]
	tsaokoarylone (**45**)	[7]
	1,7-bis(4-hydroxyphenyl)hepta-4*E*,6*E*-dien-3-one (**46**)	[33]
Diarylheptanoids	1,7-bis(3,4-dihydroxyphenyl)hepta-4*E*,6*E*-dien-3-one (**47**)	[33]
	(4*E*,6*E*)-1-(3′,4′-dihydroxyphenyl)-7-(4″-hydroxyphenyl)-hepta-4,6-dien-3-one (**48**)	[33]
	Diodiarylheptoside A–F (**49**–**54**)	[7]
	(3*S*,5*S*)-3,5-dihydroxy-1,7-bis-(4-hydroxy-3-methoxyphenyl)heptane (**55**)	[7]
	(3*R*,5*S*)-3,5-dihydroxyl-1,7-bis-(4-hydroxy-3-methoxyphenyl)heptane (**56**)	[7]
	(+)-hannokinol (**57**)	[7]
	(4′-hydroxyphenyl)-7-(4″-hydroxyphenyl)-hepta-1-en-3-one (**58**)	[33]
	Diosniponol A–B (**59**–**60**)	[33]
	(1*S*,3*R*,5*S*)-1,7-bis(4-hydroxyphenyl)-1,5-epoxy-3-hydroxyheptane (**61**)	[22]
	(1*S*,3*S*,5*S*)-1,7-bis(4-hydroxyphenyl)-1,5-epoxy-3-hydroxyheptane (**62**)	[33]
	(1*S*,3*S*,5*R*,6*E*)-1,7-bis(4-hydroxyphenyl)-1,5-epoxy-3-hydroxy-hept-6-one (**63**)	[33]
Phenanthrenes and Phenanthrenequinones	4,6-Dihydroxy-2,3,7-trimethoxy-9,10-dihydrophenanthrene (**64**)	[50]
	1-(4,7-Dihydroxy-2,6-dimethoxy-9,10-dihydrophenanthrenyl)-4,7-dihydroxy-2,6-dimethoxy-9,10-dihydrophenanthrene (**65**)	[6,50]
	7-Hydroxy-2,3,5-trimethoxy-9,10-dihydrophenanthrene (**66**)	[50]
	2,7-Dihydroxy-3,5-dimethoxy-9,10-dihydrophenanthrene (**67**)	[33,50]
	4,7-Dihydroxy-2,3,6-trimethoxyphenanthrene (**68**)	[34,50]
	3,7-Dihydroxy-2,4,6-trimethoxyphenanthrene (**69**)	[50]
	1-(2,7-Dihydroxy-4,6-dimethoxyphenanthrenyl)-2,7-dihydroxy-4,6-dimethoxyphenanthrene (**70**)	[50]
	7-Hydroxy-2,6-dimethoxy-1,4-phenanthraquinone (**71**)	[50]
	2-ethoxy-7-hydroxy-6-methoxy-1,4-phenanthraquinone (**72**)	[50]
	2-ethoxy-7-hydroxy-6-methoxy-1,4-phenanthraquinone (**73**)	[50,51]
	2,7-dihydroxy-3,4,6-trimethoxy-9,10-dihydrophenanthrene (**74**)	[51]
	Diosniposide B (**75**)	[32,52]
Phenols, Organic acids and Amino acids	Protocatechuic acid (**76**)	[6,50]
Phenols, Organic acids and Amino acids	Vanillic acid (**77**)	[6,50]
	4-Hydroxybenzoic acid (**78**)	[6,50]
	Hydroxyphenylacetic acid (**79**)	[6,50]
	Pyrocatechol (**80**)	[6,50]
	Gallic acid (**81**)	[4]
	Methyl gallate (**82**)	[4]
	Benzoic acid (**83**)	[25]
	Piscidic acid (**84**)	[36,53]
	4-hydroxy-2,6-dimethoxyphenyl-β-*D*-glucopyranoside (**85**)	[25]
	(4-hydroxyphenyl)-ethyl-β-*D*-glucopyranoside (**86**)	[25]
	Pyrocatechol 1-O-β-*D*-glucopyranoside (**87**)	[25]
	Pyrocatechol 1-O-α-*D*-glucopyranoside (**88**)	[25]
	Diosniponol C (**89**)	[32,52]
	Diosniponol D (**90**)	[32,52]
	Diosniposide A (**91**)	[32,52]
	Cyclo-(Leu-Tyr) (**92**)	[25]
	Cyclo-(Ser-Tyr) (**93**)	[25]
Flavonoids	Kaempferol-7-O-benzoate (**94**)	[37]
	Kaempferol (**95**)	[6,37]
	Kaempferol-3-O-β-*D*-glucopyranoside (**96**)	[5]
	Kaempferol-3-O-β-rutinoside (**97**)	[5,54]
	Rutin (**98**)	[6,50]
	Kushenol O (**99**)	[32]
Steroids	Ergosterolperoxide (**100**)	[37]
	β-sitosterol (**101**)	[37]
	Claucosterol (**102**)	[37]
Other constituents	(+)-Syringaresinol (**103**)	[32]
	(+)-Syringaresinol-O-β-*D*-glucopyranoside (**104**)	[5,32]
	3-Phenyl-6,8-dihydroxy-3,4-dihydroisocoumarin (**105**)	[5]
	Embran (**106**)	[25]
	Benzyl-1-O-β-*D*-glucopyranoside (**107**)	[5]
	phenylethyl-1-O-β-*D*-glucopyranoside (**108**)	[5,32]
	3′,5-dihydroxy-3,4′-methoxybibenzyl (**109**)	[5]
	4,4′-dihydroxy-3,3-dimethoxy-trans-1,2-stilbene (**110**)	[5]
	*D*-mannitol (**111**)	[5,32]
	*n*-decane (**112**)	[5]
	*N*-acetyltyramine (**113**)	[32]
	Tyramine (**114**)	[32]

**Table 3:** Summary of the pharmacological activities of *D. nipponica*, including plant parts, extract types, experimental models, and representative pharmacological effects.

Pharmacological Activity	Plant Part	Extract/Representative Active Constituent	Compound Class	Experimental Model	Main Pharmacological Findings	Ref.
Anti-inflammatory	Rhizome	TSDN; dioscin; diarylheptanoids	Steroidal saponins; diarylheptanoids	LPS-induced RAW264.7 cells; gouty arthritis rats; CIA mice; LPS-induced BV2 cells	Inhibited NF-κB/NLRP3 signaling; inhibited NO production and reduced inflammatory cytokines	[18,62]
Analgesic		Aqueous extract	Steroidal saponins	Thermal nociception and acetic acid writhing in mice	Increased pain threshold and reduced inflammatory pain	[63]
Anti-tumor		Dioscin; diosgenin ethanol extract	Steroidal saponins	HepG2, A549, osteosarcoma, colon cancer cells; xenograft models	Induced apoptosis and inhibited proliferation	[77,78,80]
Cardioprotective		TSDN; diosgenin; dioscin	Steroidal saponins	Isoproterenol-induced myocardial ischemia rats	Reduced oxidative stress and myocardial injury	[66]
Renoprotective		Dioscin	Steroidal saponins	AKI and CKD experimental models	Reduced oxidative stress, inflammation and renal injury	[8]
Anti-asthmatic		TSDN; diosgenin	Steroidal saponins	Chronic asthma mice; human airway smooth muscle cells	Suppressed IL-17A-mediated airway inflammation and improved airway remodeling	[82,101]

**Table 4:** Summary of the reported clinical applications of *D. nipponica*.

Clinical Indications	Preparations/Treatments	Core Therapeutic Effects	Ref.
Rheumatic and joint diseases	Chinese patent medicines (e.g., Chuanlong Guci Pian, Gulong Jiaonang)	Alleviates joint pain, improves physical joint function, and reduces systemic and local inflammatory responses	[105,114]
	Conventional *D. nipponica* pharmaceutical preparations		
Chronic brucellosis	Pure *D. nipponica* injection monotherapy	Relieves fatigue, joint pain and persistent fever of chronic brucellosis; improves brucellin test and serological indicators; raises short-term and long-term cure rates as an auxiliary antibacterial intervention	[107,108]
	Modified Sini San with *D. nipponica* powder (control regimen in clinical trials)		
Chronic bronchial asthma	Proprietary oral drugs (e.g., Longxiang Pingchuan Capsule, Pingchuan Yiqi Granule, Yinhuang Qingfei Capsule)	Reduces cough, sputum and asthma attack frequency; elevates FVC, FEV_1_ and PEF; relieves acute dyspnea and airway spasm; lifts total clinical effective rate and lowers long-term recurrence risk; safe for moderate-severe asthma	[110,111]
	Acupoint therapies: *D. nipponica* injection		
	Compound herbal decoction: Compound *D. nipponica* Decoction combined with Dingchuan Decoction		
Cardiovascular and metabolic diseases	Proprietary patent medicine (e.g., Di’ao Xinxuekang Capsule)	Expands coronary artery and increases myocardial blood supply; relieves angina and improves abnormal ECG; reduces postoperative cardiovascular adverse events; alleviates hyperlipidaemia and inhibits atherosclerotic plaque formation	[113,115,116]
	Single-ingredient extract tablets (e.g., Dioscornin Tablets)		
	Custom compound herbal decoctions with *D. nipponica*		

## Data Availability

The present study is a literature review, and no original experimental data have been generated. All data summarized and analyzed in this manuscript are derived from publicly available published studies, which are fully cited in the reference list.

## Abbreviations

Akt	Protein kinase B
AMPK	AMP-activated protein kinase
Bax	Bcl-2-associated X protein
Bcl-2	B-cell lymphoma 2
COX	Cyclooxygenase
ERK1/2	Extracellular signal-regulated kinase 1/2
FVC	Forced Vital Capacity
FEV_1_	Forced Expiratory Volume in one second
GC-MS	Gas chromatography–mass spectrometry
GST	Glutathione S-transferase
H&E	Hematoxylin and eosin
HO-1	Heme oxygenase-1
HPLC	High-performance liquid chromatography
IL-1β	Interleukin-1β
IL-17	Interleukin-17
Keap1	Kelch-like ECH-associated protein 1
LC_50_	Median lethal concentration
LC-MS	Liquid chromatography–mass spectrometry
LPS	Lipopolysaccharide
MAPK	Mitogen-activated protein kinase
miR-145-5p	microRNA-145-5p
MMP-9	Matrix metalloproteinase-9
NF-κB	Nuclear factor κB
NLRP3	NOD-like receptor family pyrin domain-containing 3
NO	Nitric oxide
Nrf2	Nuclear factor erythroid 2-related factor 2
PEF	Peak Expiratory Flow
PI3K	Phosphoinositide 3-kinase
Sirt5	Sirtuin 5
STAT3	Signal transducer and activator of transcription 3
TGF-β1	Transforming growth factor-β1
UPLC-QTOF-MS	Ultra-performance liquid chromatography quadrupole time-of-flight mass spectrometry
UPLC-MS	Ultra-performance liquid chromatography coupled with mass spectrometry
